# Attentional Biases toward Face-Related Stimuli among Athletes after State Thwarting Need for Relatedness

**DOI:** 10.1155/2022/2491051

**Published:** 2022-05-09

**Authors:** Na Xu, Xuchang Zhang, Xiuli Liu, Mingyu Sun, Li Rui, Yingchun Wang

**Affiliations:** ^1^School of Psychology, Beijing Sport University, Beijing 100084, China; ^2^School of Sport, Yunnan Normal University, Kunming, Yunnan 650500, China; ^3^Faculty of Education, Qufu Normal University, Qufu 273100, China

## Abstract

The present study aimed to examine attentional biases' components and processes toward the interpersonal evaluation information among athletes after state thwarting need for relatedness. 51 athletes completed a visual dot-probe task while their eye-movements were tracking. Results indicated athletes showed different attentional bias pattern. Acceptance information is early orientation (directional bias); early acceleration detection; sustained to late attention maintenance (difficulty in disengaging). Rejection information is early orientation (directional bias); early accelerated detection; continuous attention to maintenance (attention avoidance); late attention to maintenance (difficulty in disengaging). That is to say, they had motivation to seek acceptance toward the accepted interpersonal evaluation information and to avoid rejection information toward the rejected one. Therefore, it is suggested that the coaches provide more interpersonal communicating opportunities, so as to help them to restore their demands toward interpersonal communication, and provide the customized attentional bias trainings to improve their coping response after state thwarted need for relatedness.

## 1. Introduction

After Chinese Women's Volleyball Team won the World Cup, Japanese media made compliment in *volleyball weekly*. When it comes to the scene that Zhu Ting and Yuan Xinyue were hand in hand casually while entering the court, they admired the teamwork in this volleyball team as if they are beloved family. It is apparent that vital role dose interpersonal relationship play among athletes. The relationship between athletes and coaches is significant as well. After witnessing conflict between Sun Yang and his coach Zhu Zhigen as well as the blood shed of Wang Meng, we could conclude that how to deal with terrible relationship deserves more reflection. The need for relatedness is a concept in self-determination theory, which means an individual is eager to gain understanding, care, and support from interacting with others and experience a sense of belonging [1]. Early sports psychological researchers focus on how to deal with awful relationships. Researchers identified three basic psychological needs which are autonomy, competence, and relatedness needs [2]. When lacking understanding and support from teammates and coaches, athletes will generate state relatedness need thwarting after indifference, ignorance, or even exclusion [3]. The consequences of state relatedness need thwarting are as follows: excessive training, diet disorder [4], increased pressure, anxiety, and depression [5].

According to self-determination theory, when individual's relatedness needs are thwarted, their internal psychological needs balance will be broken up consequently. Aimed at recovering the initial balance, individual will generate restoration motive and try to reconnect relatedness need. This process is about relatedness need restoration [1, 6]. The study discovered that state relatedness need thwarting will push individual to generate two motivations which is to seek being accepted and avoid being rejected [7]. However, the understanding and reaction of different interpersonal evaluation information are not always the same. Researches showed that, faced with accepted information, individual with state relatedness need thwarting was more prone to favor others' suggestions and behaved more actively in interaction compared to those without need thwarting [8, 9]. Facing rejection information, individuals tend to avoid contact with community. The more contact they keep with community, the more suspicion they have. Therefore, it strengthens risks of being rejected and spurs them to generate motivations to avoid being rejected [10]. What kind of stimulus could express interpersonal evaluation information? Study showed that smiles can express not only relaxation and pleasure, but also amity and acceptance to large extent and even willingness to interact. On the other hand, angry face represents more information of being rejected and negation, standing for a threat [11].

Existing researches discovered that individuals with state relatedness need thwarting would take priority to process interpersonal clues and possessed attentional biases toward interpersonal evaluation information [10, 12, 13]. Attentional biases consist of three components which are facilitated attention, attentional avoidance, and difficulty in attentional disengaging. Facilitated attention refers to those who are easily to be attracted by some stimuli, that is to say, faster response time (RT) to probe following face pictures than to those following neutral pictures. Besides, when seeing neutral and emotional faces, participants with attentional avoidance are more prone to avoid emotional faces and remove their attention to neutral ones. Attentional avoidance means that individuals will move their attention to some opposite stimuli; when representing neutral and dangerous stimuli in the meantime, they tend to avoid dangerous ones. When individuals are attracted to some certain stimuli, it is hard for their attention to be moved to other stimuli, with slower RT to probe following face pictures than to those following neutral pictures; this is called difficulty in attentional disengaging [14]. Attention is dynamic processing procedure, which is composed of four phases: early orientation, early accelerated detection, and attentional maintenance in early stage and late period. Three components of attentional biases are playing various roles in dynamic procedure rather than antagonizing one another [15–17]. Then what kind of components of attentional biases is influenced by relatedness need thwarting? What kind of role do these components play in attentional biases processing procedure?

Relatedness need thwarting was not discriminated between trait and state in previous studies [10, 12, 13]. In fact, trait relatedness need and state relatedness need are independent mutually [18, 19]. Trait relatedness need is long-term psychologically, while state relatedness need is social exclusion generated in some specific situations and referred to as temporary effect [20]. Researches related to relatedness need thwarting pay more attention on top-down influences, for instance, self-regulation, well-being, self-evaluation, perseverance, attribution, physiological responses, mental fatigue, and so on [3, 21–23]. Earlier study is lacking in discussion about attentional processing in early stage. Attention is a procedure of stimulation and reaction, which is a temporary effect. State relatedness need thwarting happens to refer to a relatively brief expectation to potential relationship [24]. Therefore, compared to trait relatedness need thwarting, individuals with state relatedness need thwarting might have a more close connection with attention. Researchers found temporary undesirable relationship would have an impact on expected nerve processing and cognitive review by means of electroencephalography (EEG) [25]. Early cognitive process is the basis of sophisticated cognition and behavior, in which attention could be referred to as passage of transition from social perception to behavior [26]. Behavioral reaction in late stage is decided by perceptional choice in preliminary stage; therefore, discussing state relatedness need thwarting from perspective of attentional biases will contribute to making further step to explore mechanism behind this phenomenon.

What is more, previous paradigms (dot-probe task, emotional Stroop task, deployment of attention task, spatial cuing task, and visual search task) on studying attentional biases are all spatial. These paradigms predict cognitive processing before the appearance of probe point on the basis of behavioral reaction that occurred after appearance of probe point [15]. There are chiefly two shortcomings of these approaches; first of all, they could not provide dynamic procedure of attention distribution leading to difficulties in distinguishing attentional components between early stages and late ones [27]. What is more, these methods could not give an overall picture of time course characteristic in attentional biases, so it is hard to ascertain whether attentional biases occur in attentional distribution phase or evaluation phase [28]. Some scholars studied whether attentional biases took place in processes of early attention orienting or top-down processes of attention control by adopting Event-Related Potential technology (ERP). However, conclusions were not consistent as expected. Some research results found that high-anxious groups could devote more spatial attention to the recognition of intimidating messages in short period [29]. On the other side, some researches discovered that high-anxious groups put into more attentional resources on the evaluation of negative emotional stimuli [30]. Fortunately, researchers could not only learn about components of attentional biases by using ERP, but also illuminate visual attentional distribution through direct evidence from eye-tracking technology. Some researchers figured out that eye-movement technology could provide more effective index of evaluation [13]. At first, biases of saccades demonstrate attentional orientation, and first fixation latency bias could probe early acceleration, both of which were able to evaluate components of attentional facilitation. Moreover, first fixation duration bias investigated initial attentional maintenance and avoidance. At last, overall gaze duration bias evaluated overall attentional maintenance and avoidance. Three components of attentional biases have their own characteristics in different time course rather than the simple superposition of time. Diverse components could play a variety of roles in various phases of attentional processing. The mechanism and processes behind the occurrence of attentional biases are controversial in researches featuring reaction time or ERP. If possible, controversies of attentional biases could be solved through combining spatial dimension with time. Therefore, combining dot-probe task with eye-tracking technology, the present study will discuss the influence of relatedness need thwarting not only on attentional components from spatial perspective, but also on time course of attentional biases from the perspective of time.

Based on previous study, the goals of present research are twofold. First, we would like to distinguish state from trait of relatedness need thwarting. Second, we wished to investigate attentional bias components and time course toward interpersonal evaluation information of athletes with state relatedness need thwarting through combining the eye-movement tracking technique with dot-probe paradigm. Faced with admitted interpersonal evaluation information, we hypothesize that attentional pattern of athletes with state relatedness thwarting is early orientation (directional bias); early acceleration detection; sustained to late attention maintenance (difficulty in disengaging). While facing rejected one, those participants show early orientation (directional bias); early accelerated detection; sustained to late attention maintenance (attention avoidance).

## 2. Methods

### 2.1. Participants

Participants are made up of 51 athletes (ages, *M* = 21.94, *SD* = 3.20) who are in service with ages ranging from 14 to 29 years old. There are 43 male athletes and 8 female athletes with exercise experience (*M* = 4.81, *SD* = 2.73) ranging from 1 to 11 years. These athletes' titles are national first-class or above (*n* = 10), second-class (*n* = 20), and third-class (*n* = 21). Their training sports include wrestling, swimming, and basketball. The study was approved by the university's research ethics committee and volunteers provided written informed consent prior to participation.

### 2.2. Experimental Design

The experiment used a two-factor mixed experiment design with 2 (group: the state thwarting need for relatedness group, control group) ×2 (relationship clue: happy; neutral face pairs, anger; neutral face pairs). Relationship clues within subjects were tested. Dependent variables are consistency effect, RT bias score, difficulty in attention disengagement index, direction bias score, first fixation latency bias score, first fixation duration bias score, and overall gaze duration bias score.

### 2.3. Experimental Materials and Apparatus

#### 2.3.1. Cyberball Task Paradigm

Cyberball is a virtual ball-tossing computer game designed to create the experience of state thwarting need for relatedness via participant interactions with two on-screen avatars. The standard version of the task has two separate blocks, an initial inclusion block and a subsequent exclusion one. At the start of the task participants were told that they had been assigned to the Blue Team and would be playing a ball-tossing game with members of a different colored team (Verbatim instructions: Welcome to the study Blue Team member! For this part of the study, you will be playing a ball-tossing game with members of the Red Team). This manipulation was implemented to reduce in-group versus out-group uncertainty present in the use of neutral avatars and to potentially increase the real-world validity of the situation, as sudden total exclusion by an out-group is more likely than by ones in group. At the start of the inclusion block, one of the avatars passes the ball to the player who then makes a selection, via a button box, to pass that ball to either one of the two avatars. In the initial inclusion block, upon receiving the ball the selected avatar would then either (1) pass the ball directly back to the player (50% chance), (2) pass the ball to the second avatar who would then pass the ball directly back to the player (40% chance), or (3) pass the ball to the second avatar who would then pass the ball back to the first avatar who would then pass the ball directly back to the player (10% chance). This sequence of events was repeated for a total of 40 passes, 26 of which were target trials which involved the player monitoring another avatar. After these 40 exchanges had accrued, the exclusion block began after a 10 s rest break. In this block, the first 8 passes proceeded in the same manner as in the preceding inclusion block after which the exclusion trials began. After this point the avatars ceased passing the ball to the player and proceeded to pass the ball back and forth between themselves, never passing the ball to the player, until a total of 24 passes had been made. At this point the participant was told that the task had been completed.

#### 2.3.2. Questionnaire for Operation and Check

The questionnaire of state relatedness need thwarting for operating and checking was adapted [22], to monitor situational setting of thwarting need for relatedness. This one has been applied in the field of sports [31], including pleasure, popularity, sense of belonging, and time spirit experienced during playing sports with others. The items in the questionnaire were rated from 1 (“barely nothing”) to 7 (“too much”). In present research, the Cronbach coefficient of questionnaire was *α* = 0.88.

#### 2.3.3. The Positive and Negative Affective Schedule (PANAS)

This research adopted PANAS scale [32], measuring participants' emotions after experiencing state relatedness need thwarting. In this study, the Cronbach coefficient of questionnaire was *α* = 0.75.

#### 2.3.4. State Relatedness Need Thwarting Questionnaire

This paper adopted the Basic Psychological Needs Scale (BPNS) compiled by Richer, which is translated and revised by domestic scholar Kong [33]. The present study selected one dimension—state relatedness need thwarting from scale, which was made up of 5 items, for instance, “I thought others would despise me.” Seven-point Likert scale was adopted, ranging from 1 (“not at all like me”) to 7 (very much like me). The higher scores you gain, the more likely you are going to experience state relatedness need thwarting. This subscale has been applied by researchers many times in the field of sports [34]. In current study, the Cronbach coefficient of scale was *α* = 0.85.

#### 2.3.5. Experimental Apparatus

This research was conducted by means of TobiiT60XL tracker. The participants need to be seated approximately 65 cm in front of a 24-inch, 60-Hz monitor with resolution of 1920 ∗ 1600 pixel in a low sun-shining environment.

#### 2.3.6. The Dot-Probe Task

**Figure 1:**
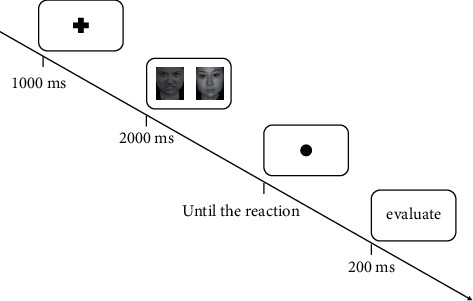
The dot-probe task.

Attentional bias was tested by dot-probe task set on basis of Zhang et al.'s [35] research. There are 10 trials for exercise and 108 formal trials. Participants were required to complete the dot-probe task. The photographic stimuli is composed of 20 female and male happy faces, 20 female and male angry faces, and 40 neutral faces. These facial pictures were adapted from Chinese Affective Picture System (CAPS) [36]. Two different pictures were presented twice. At the beginning of each single trial, a fixation cross was presented in the center of the screen for 1000 ms to prepare participants for the following procedures. Then, random picture pairs were immediately displayed for 2000 ms, followed by replacement of one of the pictures by a black dot (•). Each picture pair was presented twice so that each picture location was counterbalanced by presenting them on both sides of the computer screen twice. When the fixation cross was presented, participants were asked to have to fixate the cross, viewed subsequent pictures, and pressed one key (*A*) when the dots were located on the left side of the screen and another key (*L*) when the dots were on the right side as quickly as possible. Each probe appeared until a response was made, followed by a blank screen for 200 ms. In total, 108 trials were performed in this experiment (seen in Figure 1).

### 2.4. Procedure

At first, participants needed to fill in an informed consent form and state relatedness need thwarting questionnaire. Subsequently, participants were divided into groups through Cyberball task at random and finished questionnaire for operation and check as well as PANAS scale. Afterwards, participants were required to rectify eye-movement system and completed the dot-probe tasks at the same time. At last, participants were informed of experimental device. Researchers covered the remuneration and expressed gratitude.

## 3. Results

### 3.1. The Operable Examination of State Relatedness Need Thwarting and the Impact of Need Thwarting on Emotions

The independent variable is group; scores of operable examination questionnaire and positive as well as negative emotions are dependent variable. The independent sample *t*-test analysis resulted in that the situational setting of state relatedness need thwarting is effective (*t* (1, 49) = 5.21, *p* = 0.000, cohen's d = 1.459). Besides, the Cyberball task could not cause variance between positive emotions (*t* (1, 49) = 1.53, *p* = 0.13, cohen's d = 0.428) and negative emotions (*t* (1, 49) = -1.57, *p* = 0.12, cohend's = −0.440).

### 3.2. RT Data

Based on Gladwin et al.'s research [11], data which was under 200 ms and above 2000 ms and extreme data which was above ± standard deviation were eliminated. Using the trait thwarting need for relatedness as covariate, the analysis of covariance with 2 (group of state relatedness need thwarting, control group) ×2 (happy; neutral face pairs, anger; neutral face pairs) with slope homogeneity test resulted in that interaction effects among all variables and the trait thwarting need for relatedness were not significant. Therefore, the analysis of covariance could be carried out. Results indicated that consensus was not significant, *F* (1, 49) = 0.549, *p* = .459, *η*_*p*_^2^ = .001, that is to say, no inhibition of return or consensus facilitating effect. The results demonstrated a significant main effect for groups, *F* (1, 49) = 16.702, *p* = .040, *η*_*p*_^2^ = .021, and other main effects as well as interaction effects were not significant. In the dimension of RT bias score, main effect of facial types was significant, *F* (1, 49) = 1.134, *p* = .289, *η*_*p*_^2^ = .011. Besides, the post hoc test revealed that the bias scores of angry faces were greater than those of happy faces. The analysis of the difficulty in attention disengagement index showed a significant main effect in groups, *F* (1, 49) = 9.558, *p* = .003, *η*_*p*_^2^ = .089. In addition, the post hoc test demonstrated that the difficulty in attention disengagement index of groups of state relatedness need thwarting was more significant than control groups (seen in Table 1).

### 3.3. Eye-Movement Data

According to definition of fixations from Akeju [37], fixations in each trial were effective only if the following two conditions occurred: (i) Participants fixated in the central region (fixation cross) before picture onset; (ii) saccades kept stable within a 1° visual angle for 100 ms or longer and first fixation latency lasted for 100 ms or longer. Those would be eliminated if effective trials were under 40%; as a result, 42 effective participants were chosen.

#### 3.3.1. Examination of Attention Bias Components

The one-sample *t*-test analysis showed that the attention biases of participants with state relatedness need thwarting toward happy faces were attentional facilitation; attentional maintenance; difficulty in attention disengagement. In first fixation direction bias score, *t* (1, 20) = 2.079, *p* = 0.050, cohend's = 5.444 (scores are above 50). In first fixation latency score, *t*（1, 20） = -5.941, *p* < 0.001, cohend's = −1.267 (scores are less than 0). In first fixation duration bias score, *t* (1, 20) = 2.921, *p* = 0.008, cohend's = 0.623 (scores are above 0). In overall gaze duration bias score, *t* (1, 20) = 18.651, *p* < 0.001, cohend's = 3.976 (scores are above 50).

The attention biases of participants with state relatedness need thwarting toward angry faces were attentional facilitation; continuous attention to maintenance (attention avoidance); difficulty in attention disengagement. In first fixation direction bias score, *t* (1, 20) = 3.512, *p* = 0.002 cohend's = 5.817(scores are above 50). In first fixation latency score, *t* (1, 20) = −7.082, *p* < 0.001, cohend's = −1.510 (scores are less than 0). In first fixation duration bias score, *t* (1, 20) = −3.356, *p* = 0.003, cohend's = −0.716 (scores are less than 0). In overall gaze duration bias score, *t* (1, 20) = 20.088, *p* < 0.001, cohend's = 4.283 (scores are above 50).

#### 3.3.2. Examination of Attention Bias Score Variation

We used 2 (group of state relatedness need thwarting, control group) ×2 (happy; neutral face pairs, anger; neutral face pairs) repeated measures covariance analysis of the four EM indices with the trait thwarting need for relatedness as covariate. Slope homogeneity test resulted in that interaction effects among all variables and the trait thwarting need for relatedness were not significant. Therefore, the analysis of covariance could be conducted.

The results in first fixation direction bias score demonstrated a significant main effect for group, *F* (1, 40) = 9.497, *p* = 0.003, *η*_*p*_^2^ = 0.102. The post hoc test showed that the first fixation direction bias scores of group of state relatedness need thwarting were greater than those of control group. The results in first fixation latency score revealed a significant main effect for group, *F* (1, 40) = 19.339, *p* = 0.000, *η*_*p*_^2^ = .195. The post hoc test showed that first fixation latency scores of group of state relatedness need thwarting were more significant than those of control group. The main effect for facial type was significant, *F* (1, 40) = 6.307, *p* = 0.014, *η*_*p*_^2^ = 0.073, presenting that absolute value of first fixation latency scores of angry faces was more significant than those of happy ones. In first fixation duration bias score, main effect for facial type was significant, *F* (1, 40) = 10.236, *p* = 0.002, *η*_*p*_^2^ = 0.113, manifesting that absolute value of first fixation duration bias scores of angry faces was more significant than those of happy ones. In addition, interaction effect was significant, *F* (1, 40) = 12.106, *p* = 0.001, *η*_*p*_^2^ = 0.131. Simple effect analysis found that first fixation duration bias score of state relatedness need thwarting group toward angry faces was more significant than control group, *p* = 0.002; that is to say, the initial attentional time of state relatedness need thwarting group toward angry faces was greater than control group. Besides, first fixation duration bias scores of happy faces in group of state relatedness need thwarting were more significant than those of angry faces, *p* = 0.000. The results in overall gaze duration bias score demonstrated a significant main effect for group, *F*(1, 40) = 28.264, *p* = 0.000, *η*_*p*_^2^ = 0.261. The post hoc test showed that overall gaze duration bias scores of group of state relatedness need thwarting were more significant than those of control group (seen in Table 2).

## 4. Discussion

### 4.1. Focuses on Two Dimensions of Attention Bias: Space and Time

Recent studies pointed out that research paradigms of attention bias could be divided into spatial dimension and time one [15]. Dot-probe paradigm is the main method to investigate attentional biases from the perspective of space. Eye-movement technology is a vital and practical method in disclosing cognitive processing characteristics and time course of attention bias. Present research studied how state relatedness need thwarting influenced athletes' attention bias components in interpersonal evaluation information and characteristic of time course by combining eye-movement tracking technology and dot-probing task on basis of two dimensions.

From perspective of space, dot-probing data demonstrated that athletes with state relatedness need thwarting showed early orientation and difficulty in attention disengagement to angry faces, with merely difficulty in attention disengagement to happy faces. Results of current experiment are effective according to findings in RT analysis of congruence effect. However, it is hard to deduce the whole processing pattern of attention bias merely depending on variations in RT. Combined with the dimension of time, eye-movement results demonstrated what follows: (i) Attention pattern of happy faces turned out to be early orientation (directional bias); early acceleration detection; sustained to late attention maintenance (difficulty in disengaging), (ii) whereas attention pattern of angry faces was early orientation (directional bias); early accelerated detection; continuous attention to maintenance (attention avoidance); late attention to maintenance (difficulty in disengaging). The attention pattern of accepted interpersonal information is consistent with original hypothesis; when it comes to the pattern of rejected one, the hypothesis is confirmed partly. This result is in line with Chen et al.'s research [13]; that is to say, state relatedness need thwarting could generate attentional bias in interpersonal evaluation information about both acceptance and rejection. Current research found furthermore, from perspective of time, attentional bias of state relatedness need thwarting is a process which consists of orientation, accelerated detection, and maintenance. Besides, attentional bias differs in accepted and rejection information.

Athletes with need thwarting would adopt various strategies in attentional biases processing procedure. Firstly, after experiencing state relatedness need thwarting, participants spent more time on locations of emotion-related stimuli. Secondly, when spatial vision disappeared without picture stimuli from emotional faces, thes athletes were more prone to return to where stimuli occurred, thus resulting in more RT [38]. Traditional dot-probing task is inclined to deduce attention bias through comparing RT of emotional face stimuli and neutral ones. The present research avoided limitations of single spatial method and got more persuasive results. Research showed how participants' attention bias generated, developed, and changed after thwarting their state relatedness need from two perspectives and provided proof about how each time phase of different interpersonal evaluation relationship affected attentional bias. This study discovered that, under state relatedness need thwarting, first fixation duration bias score revealed a significant interaction effect; the need thwarting group's first fixation duration bias was more significant than control group; the need thwarting group's bias score of happy faces was higher compared to those of angry faces; first fixation duration bias score was greater than zero when seeing happy faces, but less than zero after facing angry ones. These results demonstrated that original attentional maintenance existed in both accepted and rejected information; then, the former showed difficulty in attention disengagement, and the latter revealed attentional avoidance. Anyway, this article dived into attention bias of state relatedness need thwarting comprehensively by combining eye-movement tracking technology with dot-probing task based on space and time.

### 4.2. Athletes with State Relatedness Need Thwarting Response Accepted and Rejected Information with Various Attention Bias Mechanism

Overall, this study balanced trait relatedness need thwarting; athletes paid more attention on accepted information rather than rejection information. In overall gaze duration bias, scores of state relatedness need thwarting group toward interpersonal evaluation information were more significant than control group. And when faced with happy and angry faces, the overall gaze duration bias scores were all greater than 50. However, there was a significant interaction effect in first fixation duration bias score. Facing happy faces, first fixation duration bias score was greater than 0, whereas facing angry ones, score was less than 0. All in all, participants with state relatedness need thwarting had attentional maintenance no matter facing accepted information or rejection one. But, difficulty in attention disengagement existed in the process of accepting accepted information all the way. There were both difficulty in attention disengagement and attentional avoidance in rejecting rejection information.

Based on the goal conflict model [39], when state relatedness need was thwarted, people were eager to recover this need, generating strong motivation to seek for connection. When people with state relatedness need thwarting happened to meet accepted information, they would be attracted by accepted stimuli easily with attention hardly transferring to other stimuli. On the contrary, when faced with rejection information, they were prone to avoid dangerous stimuli and transfer their attention to neutral or opposite stimuli, therefore generating attentional avoidance [14]. The present study discovered that bias scores of happy faces in group of state relatedness need thwarting were more significant than those of angry faces; therefore, compared with accepted information, participants with state relatedness need thwarting paid less time on rejection information with less attentional biases.

In addition, on the basis of dual-system model, participants' processing of rejection information was influenced by reflective and impulsive systems [40, 41]. Impulsive system was used to consider whether this need could be accepted again, while reflective system was to evaluate whether this need could be rejected again in the accepted process. These two systems are mutually controversial and competitive, so when facing rejection information, participants with state relatedness need thwarting would generate two kinds of attentional biases which are attentional avoidance and difficulty in attention disengagement.

Different interpersonal evaluation information has various impact on attentional biases and influences how athletes deal with state relatedness need thwarting. Therefore, coaches could construct platforms for athletes to recover state relatedness need, provide more opportunities for team members to communicate, and set up common objectives so as to strengthen mutual trust and raise team recognition. Coaches could also draw up targeted plan to train attentional biases in order to enhance athletes' capability to cope state relatedness need thwarting.

## 5. Conclusion

When athletes' state relatedness need thwarted, they are more prone to generate more attentional biases toward accepted information compared to rejection information. They would generate motivation to accept acceptance of accepted information and generate motivation to reject rejection information.

## Figures and Tables

**Table 1:** The covariance analysis results of various faces toward RT and RT bias score (*N* = 51).

	Attention bias score			RT bias score			The difficulty in attention disengagement index		
	*F*	*p*	*η*_*p*_^2^	*F*	*p*	*η*_*p*_^2^	*F*	*p*	*η*_*p*_^2^
Groups	16.702	0.000^*∗∗*^	0.040	0.138	0.711	0.001	0.138	0.711	0.001
Facial types	1.399	0.238	0.003	17.123	0.000^*∗∗*^	0.149	17.123	0.000^*∗∗*^	0.149
Groups × Facial types	0.235	0.628	0.001	1.134	0.289	0.011	1.134	0.289	0.011

**Table 2:** The covariance analysis results of four EM indices among various groups (*N* = 42).

	First fixation direction bias			First fixation latency bias			First fixation duration bias			Overall gaze duration bias		
	*F*	*p*	*η*_*p*_^2^	*F*	*p*	*η*_*p*_^2^	*F*	*p*	*η*_*p*_^2^	*F*	*p*	*η*_*p*_^2^
Groups	9.497	0.003^*∗∗*^	0.102	19.339	0.000^*∗∗*^	0.195	1.1632	0.284	0.014	28.264	0.000^*∗∗*^	0.261
Facial types	1.764	0.188	0.021	6.307	0.014^*∗*^	0.073	10.236	0.002^*∗∗*^	0.113	0.282	0.597	0.004
Groups × Facial types	0.003	0.958	0.000	0.002	0.965	0.000	12.106	0.001^*∗∗*^	0.131	0.520	0.473	0.006

## Data Availability

The data used to support the findings of the study are available from the corresponding author upon request.
